# Escaping Historical Lock-in—Redesigning Wastewater Treatment Plants and Their Microbiomes for the 21st Century

**DOI:** 10.1021/acs.est.5c06208

**Published:** 2025-07-15

**Authors:** Willy Verstraete, Laurence Strubbe, Ilje Pikaar, Tom W. Vinestock, Po-Heng Lee, Silvio Matassa, James Chong, Jizhong Zhou, Glen T. Daigger, Miao Guo

**Affiliations:** † Centre for Microbial Ecology and Technology, 26656 Ghent University, Ghent 9000, Belgium; ‡ Eawag, 28499 Swiss Federal Institute of Aquatic Science and Technology, Dübendorf 8600, Switzerland; § School of Civil Engineering, 1974 The University of Queensland, Brisbane, QLD 4072, Australia; ∥ Department of Engineering, Faculty of Natural, Mathematical & Engineering Sciences, 4616 King’s College London, London WC2R 2LS, United Kingdom; ⊥ Department of Civil and Environmental Engineering, 4615 Imperial College London, London SW7 2AZ, United Kingdom; # Department of Civil, Architectural and Environmental Engineering, 9307 University of Naples Federico II, Naples 80125, Italy; ∇ Department of Biology, University of York, York YO10 5DD, United Kingdom; ○ Institute for Environmental Genomics (IEG), School of Biological Sciences, University of Oklahoma, 101 David L. Boren Blvd., Norman, Oklahoma 73072 United States; ◆ 1259 One Water Solutions, 9396 Arbor Court, Plymouth, Michigan 48170, United States; □ Department of Civil and Environmental Engineering, College of Engineering, University of Michigan, 1351 Beal Avenue 177 EWRE Building, Ann Arbor, Michigan 48109-2125, United States

**Keywords:** WWTP, microbiome, wastewater, portable water, resource recovery, optimization, machine learning

## Abstract

Wastewater treatment plants (WWTPs) have gradually, over the last hundred years, been designed and extended to deal with a sequence of problems, including a) odor, b) suspended solids, c) organics, d) ammonia, e) nitrate and phosphate, and f) recalcitrant pollutants. The line of historical developments was piecemeal rather than holistic and did not focus on sustainability, resource recovery, and water reuse. On the contrary, microbial processes that accelerated the removal of nitrogen were incorporated and heralded as a positive part of the “cleanup” agenda, despite their relatively large energy consumption and substantial production of nitrous oxide, a potent greenhouse gas. The time has come to examine the historical, technological, and microbiological lock-in present in today’s WWTPs, so that a more coherent integrated system can be developed for future generations. Some disruptive strategies are outlined, and a categorization of processes in terms of their potential for the future is formulated.

## Graphical abstract

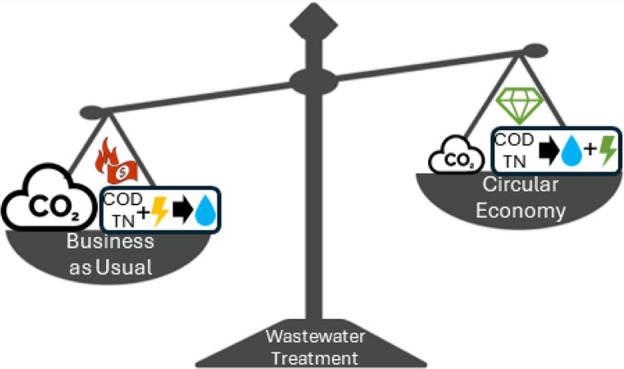

## Overall Platform

Wastewater treatment plants (WWTPs) are essential for protecting human health and the environment. Over the last hundred years, WWTPs have tackled a sequence of problems: from odor control to organic matter and nutrient removal, and more recently to meeting legislative requirements for the removal of micropollutants. However, while wastewater contains a range of valuable resources, resource recovery has historically not been a primary objective.

Resources, such as water, energy, and nutrients, mainly phosphorus (P), are becoming increasingly scarce, making the release of valuable organic materials and nutrients into the environment—often as greenhouse gases—unsustainable. This practice also wastes energy and fails to promote efficient resource management. The growing urgency of climate change and the global push for investment in green energy, energy storage, and resource recovery legislation provide a timely opportunity to rethink WWTPs.

Rethinking WWTPs in light of new technological possibilities and novel insights into the potential and limitations of microbial communities should prioritize resource recovery and reuse. However, the success of resource recovery and reuse depends on consumer acceptance. To gain consumer acceptance, products generated by WWTPs must reliably meet stringent hygiene standards, be cost-effective, and address the concerns of society and each individual as a consumer. Various experiences show that these two issues—hygiene and cost—are indeed at the forefront of the public’s perception regarding resource recovery.

Achieving these multiple objectives requires a process with a resilient microbiome, ensuring reliable treatment performance. Note that the microbiome is a mixture of bacteria, eukaryotic microorganisms, and archaea, although the latter two also play a role in resource recovery; the paper focuses mainly on the role of bacteria. Understanding the role of bacteria in this context, along with its limitations and opportunities, is essential for advancing three key types of recovery products: potable water, energy, and nutrients.

## Potable Water from Wastewater

At present, WWTPs generally produce a “half-fabricate”, water that can be safely and legally discharged into the environment. The reasoning is that “Mother Nature” then achieves further cleanup without any additional costs. What is discharged can be extracted to make drinking water available a short distance down the river; this raises little or no public concern. The *concept is practiced in the so-called A-B process;* while water for nonpotable purposes is common,^1^ the direct conversion of wastewater to potable water has historically been rarely practiced. However, this situation is changing, primarily because of water scarcity. This is particularly visible in places such as the Southwestern United States, where a significant number of potable reuse projects are progressing, in addition to the Orange County Water District project^2^ and the initiatives in Singapore.^3^ One noteworthy historical example is Windhoek, Namibia, the most arid country in Sub-Saharan Africa, where direct potable reuse has been required since 1968 to secure water supply to the city.^4,5^ With regard to Europe, since the year 2000, at the Aquaduin plant, Oostduinkerke, Belgium,^6^ domestic wastewater leaving the activated sludge plant and subsequently undergoing membrane filtration and dune infiltration is delivered as drinking water to consumers, with more than half of that drinking water originating from wastewater. This technology has been established for several decades now. Moreover, there are several locations where canal water, composed mainly of effluents discharged from WWTPs, is upgraded by membrane technology to drinking water, such as the Farys plant in Ostend, Belgium.^7,8^ These examples of both direct and indirect potable water reuse have shown that these operations are technically feasible and cost-effective. Moreover, these technological advances are supplemented by an increased understanding of how to interact with the public to gain acceptance of direct potable reuse.^9–11^ Yet, balancing the environmental urgency and economic benefits of potable reuse remains a key trade-off.

Closing the circle in this way is a great achievement. The microbiomes involved are those we study at present, such as activated sludge communities in WWTPs, biofilms in sand infiltration dunes used for drinking water production (biofiltration),^12^ and populations of bacteria and microeukaryotes found in the distributed drinking water.^13^ There is plenty of room for further in-depth analysis of these microbiomes, their integration into a complete process train (i.e., from wastewater to drinking water),^14^ and of ways to guarantee and monitor stable performance.

## Energy from Wastewater

The public is aware that energy consumption drives climate change. However, the knowledge that approximately 4% of current fossil fuel-based energy is used for the treatment of domestic wastewater^15^ is not yet a cause of popular concern. This may change in the near future due to increasing transparency about the energy system and the environmental technology required to secure popular consent for the energy and sustainability transition.

Today, WWTPs consume energy, mainly for aeration.^16^ The energy present in the water as low-temperature heat,^17^ as organics (i.e., expressed as chemical oxygen demand (COD), and as ammonia (NH_3_) is only occasionally and partially recovered.^18^ The most developed energy recovery process is anaerobic digestion (AD) of the organics. This route is usually applied indirectly; the harvested organics (primary and secondary sludges) are subjected to AD and represent a recovery of 10–14% of the energy originally present.^17^ The direct treatment of wastewater by AD has been explored for several decades but is hampered by the low level of COD and, particularly in temperate regions, by the relatively low temperature of wastewater, coupled with the solubility of methane in the treated effluent.^19^ The potential energy recovery by direct treatment is of the order of 0.25 kWh/m^3^.^20^ Overall, in the context of historically locked-in WWTPs, AD is fitting, but if we consider the overall WWTP energy content, it is by no means significant.

The microbiomes related to anaerobic bioconversion of organics into usable products have been heavily explored over the past few decades in terms of metabolic fluxes and taxonomic compositions. Fatty acids—short and elongated—could represent a type of recovery, but their origin imposes a heavy burden on their subsequent value chain. Clearly, the winning product is the production of methane gas as a reusable resource because it is well accepted, has large, established markets for this fuel, and can often be used directly on-site.

In terms of optimizing biomethane production, despite ongoing research, the management of biomethane-producing microbiomes is thus far limited to controlling the physical and chemical boundary conditions. So far, no breakthroughs in the efficiency of methane production have been achieved through microbiome engineering. The recent discovery of oxygenic photosynthetic bacteria^21^ significantly taking part in the methane cycle sheds light on reinforcing biomethanation while combining it with solar energy storage.^22^

## Nitrification/Denitrification

The question of why approximately 50% of the energy consumed in WWTPs is utilized by aerobic heterotrophs and nitrifiers^16^ is rarely addressed. Nitrification and denitrification are widely embraced as essential, but they should more appropriately be considered a kind of inefficiency: they consume energy directly through aeration and indirectly through COD consumption. In addition, these processes produces substantial amounts of greenhouse gases both directly and indirectly.^23^ The nitrous oxide (N_2_O) generated, mainly due to the chemical instability of the metabolites, accounts for more than 22% of the total GHG emissions from both aerobic and anaerobic treatments.^24^ In relation to sustainability, it is crucial to note that NH_3_ from wastewater can be used as an energy source.^25^ NH_3_ could be decomposed with or without organic compounds via reformation through solid oxide fuel cells.^26^ NH_3_ has a heating value of 5.3 kWh kg^–1^ similar to biogas (6.1 kWh kg^–1^) and it could cofuel existing biogas and biomethane turbines for combined heat and power generation or be exploited for high-efficiency electricity production in emerging electrochemical fuel cells.^25^

The decision to include urine in the wastewater stream has influenced the evolution of wastewater treatment to its current stage. This was done in the mid-19th century in the industrializing world^27^ for reasons of convenience and because nitrogen and phosphorus discharges were not considered as a problem, even though they represented a significant loss of nutrient resources. Today, however, there is a lot of research into urine separation.^28^ Although urine represents less than 1% of the volume of wastewater, it contains 80% to 90% of the nutrients.^29^ Early urine separation, instead of the current end-of-pipe solution, would significantly change wastewater management, as the treatment plant would only need to focus on extracting energy from organic matter and from NH_3_ (see below).

The microbiology related to nitrification continuously develops and indicates that the nitrifiers are very competent creatures, constantly evolving in terms of their capabilities to operate under various and variable environmental conditions and to cooxidize plenty of molecules. The question of minimizing and preferably fully excluding the activity of nitrifiers has so far been rarely addressed. Regulating microbiomes has proven to be difficult, but in terms of nitrification, some remarkable alterations in electron flow have been possible by implementing mechanisms such as bioanode ammonium oxidation and electro-anammox.^30,31^ These approaches warrant further attention.^32^

## Nutrients and Other Commodities from Wastewater

The current technology of wastewater treatment has the potential to recover nutrients such as nitrogen in the form of NH_3_ and phosphorus in the form of struvite and vivianite from water.^33,34^ These recovered nutrients are mainly proposed for use as fertilizers, although other applications are also being explored. Research attention has also been directed toward exploring the recovery of paper-based cellulose^34^ and microbial-based polymers such as PHA (polyhydroxyalkanoates) and extracellular polymeric substances (EPS).^34,35^ The major challenge lies in the willingness of consumers to use such recovered products. Existing market players, who rely on traditional, economically optimized production methods, such as the extraction of raw materials and the manufacture of products from primary or unprocessed resources, continue to push back against the use of products recovered from the wastewater industry. Moreover, the presence of recalcitrant compounds, such as PFAS, pharmaceuticals, and microplastics, in these recovered products raises significant sustainability concerns.

Enhanced biological phosphate recovery (EBPR) depends on the availability of readily biodegradable organic matter during the anaerobic phase. However, this approach has several drawbacks. First, it consumes organic matter that could otherwise be used to produce biomethane, representing a missed opportunity for energy recovery. Second, incorporating an anaerobic phase increases the treatment plant’s footprint, which can pose spatial and economic challenges. Third, the efficiency of biological P removal is constrained by the concentration of biomass, often necessitating chemical precipitation to meet stringent phosphorus effluent limits. The perspective for P recovery from wastewater lies in further exploring the bioleaching of P from ash after the sewage sludge has been incinerated.

Recently, the insight has risen that biosolids could be technically and economically part of Carbon Capture and Storage. Processes such as torrefaction/pyrolysis can yield biochar, which is usable as a sorbent in the treatment of water. Moreover, the stabilized biosolids, provided appropriate legislative adjustments in relation to material properties, can serve as a filler in certain construction materials. The latter approaches are interesting in the context of storing the carbon present in the biosolids in a form that gains a new life and function, rather than combusting it directly to CO_2_.^36^

Despite the abovementioned limitations, the microbial processes involved in recovery processes such as EBPR, and the production of PHA and EPS commodities, have experienced remarkable progress in terms of scientific insights and overall control technology. However, microbial biotechnology and advanced microbial ecology tools have not so far been able to turn these products into “must-haves” for the consumer.

## Rethinking in Terms of Recovery: What Should Be Handled by the Microbiome?

It is essential to dare to question the need for microbiology in the treatment of wastewater. Indeed, several attempts have been made to design a fully nonbiological approach.^37^ The studies of the plant in Wilp, The Netherlands, tend to indicate that a process line with only a high-rate bioassimilation step, complemented with physical–chemical steps, is likely to achieve similar process and energy efficiencies to the conventional biological approach. These facts, although preliminary, must be taken into account.

The corollary of the development of nonbiological processes is the fact that biotech processes should focus on the positive power of biology, while negative bioprocesses should be excluded. Concretely, this comes down to increasing the use of bioassimilation, improving the methane production component, decreasing the use of nitrification/denitrification and biological phosphate removal, and steering away from the belief in various other, undesired and poorly accepted, recovery products (i.e., recovered N and P as fertilizers for agriculture and microbial-based polymers).

In practice, to maximize energy recovery from anaerobic digestion, the organics should not be converted to activated sludge biomass, which is then starved and rendered difficult to convert in the subsequent AD process. Instead, they should be harvested directly as far as possible. In addition, the microbial biomass should be kept as “young and energy rich” as possible. *Note that the microbiome is a mixture of bacteria*.^38^ In the “A” or assimilation step, organic matter is removed by sorption onto the activated sludge flocs.^39^ The organics are then subjected to AD. This way, much less influent organic carbon is subjected to energy-intensive aerobic conversion and oxidation. The net gain (methane over recovered organic matter in wastewater) from a well-designed A-step is on the order of 35%.^40^

The microbial aspect of “assimilation” of organics in general, and nutrients in particular, into cellular biomass—which can readily be separated and subsequently digested in concentrated form—is full of challenges. Particularly intriguing is the recent finding that the involvement of microeukaryotes can possibly provoke higher Carbon Use Efficiencies (CUEs).^41^ The question of which groups of microorganisms to exploit for maximal separation of C, N, and P upfront in an “assimilation”-focused treatment step is an important one in this line of thinking.

It is evident that in an optimized “A” step, the amount of nitrification should be kept minimal. Approaches to inhibit nitrifiers have been studied in agricultural soils (e.g., the concept of biological nitrification inhibition by plants),^42^ but so far, they have not been proven effective. Predators and parasites of nitrifiers have been described,^43^ but within wastewater treatment, it is more reasonable to focus on outcompeting nitrifiers while promoting ammonia-assimilating organisms.

By maximizing the capture of C, N, and P into particulates that go for AD, the latter process really gains importance. A million-dollar question is: is it possible to select for microbiomes with more anabolism, higher overall cell yields, and more effective ways to capture C, N, and P?

At present, the treatment of digestates is a major challenge and a nuisance for practitioners. It is imperative to make this “bottom end” simpler. NH_3_ can be stripped, but it must be recognized that the demand for such wastewater-derived NH_3_ is very low. Hence, the proposal is to focus on the valorization of recovered NH_3_ as a source of energy. Thermal cracking of NH_3_ to hydrogen and nitrogen gas has been proposed;^44^ methods to electrochemically convert NH_3_ to hydrogen and nitrogen gas have also been explored.^45^ These processes, focusing on ammonia as a zero-carbon energy vector,^25^ should receive high attention because they can provide a sustainable form of valorization of the residual energy present in the NH_3_ molecule that can be directly used on-site.

As far as the recalcitrant organics and the minerals trapped in the organic matrix are concerned, the obvious line of further treatment is the separation of the solids and their incineration. These processes are outside the scope of biotechnology; they generate ashes rich in important minerals, not only P but also rare earth elements. However, it could be that in the future, the selective leaching of minerals by bioprocesses from ashes may offer new potential for the recovery of these minerals.

One of the microbial carbon transformations of interest in recovery is chain elongation, a metabolic process that involves stepwise carbon chain elongation of short-chain organic compounds, such as C1–C5 short-chain carboxylic acids, into larger, more complex organic molecules, such as C6–C8 medium-chain carboxylic acids (MCCAs) via reverse oxidation pathways. Microbial chain elongation has been observed in both natural environments, such as soil, and engineered systems, such as waste-resource recovery systems. Chain elongation has been used for groundwater bioremediation of trichloroethene, longer chain alcohol (C4–C8) production for biofuels, and carboxylates as high-added-value chemicals.^46,47,48^ Yet, as indicated above, for applications outside the “combustion sector”, the origin of these fermentation products poses a heavy burden on their subsequent value chain; cultural and religious aspects are very powerful and need to be taken into consideration.

There remains one extra point of attention: the removal of residual levels of recalcitrant molecules. So-called “Forever Chemicals”, such as PFAS, microplastics, toxic metals, and residues from pharmaceuticals, and personal care products tend to slip through the “rationally designed” WWTP and need to be dealt effectively at the end of the water treatment process. In the overall concept of sustainability, the recovery of reclaimed water, energy, and nutrients is a goal that must be set as central. Yet, since the entry of nonbiodegradable molecules into wastewaters is inevitable, techniques that remove these molecules (particularly membranes and sorption of these usually polar molecules on activated carbon or ion exchange resins) and that, moreover, destroy them fully (particularly fragmentation by ozonation and incineration) will and must be an intricate part of the adequate treatment of wastewater. These current approaches have a heavy environmental footprint. The Life Cycle Analysis aspects of sorption of polar pollutants, including the breakdown products of such pollutants, need to be scrutinized.

Compared to physical and chemical approaches to removing recalcitrant compounds, using appropriate biotechnology that bind these recalcitrant compounds into highly adsorbent biomass may offer a significantly lower carbon and energy footprint. An example is the sorption of PFAS on Gram-negative microbial cells.^49^ Clever bioalternatives, such as trapping these molecules in larger humus-like molecules by biobased humification processes or removing them using microbial communities empowered by elegant biocatalysts such as manganese-oxidizing bacteria, could offer new strategies and processes that increase the sustainability and overall energy efficiency of WWTPs for future generations.

Rethinking WWTPs with a focus on resource recovery requires not only a thorough understanding of the microbiome’s role but also careful consideration of the most valuable recovery product for the specific location. Pursuing multiple recovery objectives simultaneously may not be feasible; prioritization is key to maximizing both efficiency and impact.

## Process Integration, Modeling, and Optimization

The design of wastewater treatment and resource recovery plants is a complex, multistage process synthesis problem involving multiple objectives and many constraints. While WWTP technologies have traditionally been selected based on the expert knowledge and experience of practitioners, as well as simplifying assumptions,^50^ this approach to design can be conservative, slow to adapt to new technologies or changing priorities, and rarely results in a fully optimized plant.

Modeling and integrated assessment with mechanistic models have been proven to significantly improve the design and operation of wastewater treatment and resource recovery plants by providing a structured framework for optimizing complex processes.^51^ The Benchmark simulation model 2, for example, allows for a holistic approach to optimization, with integration of the various treatment steps, leading to improved end-to-end performance. Interactions between different unit processes, such as primary and secondary clarifiers, activated sludge reactors, and anaerobic digesters, can be modeled and considered.

Equally, the scale-up of new treatment and recovery options, from lab scale to pilot and industrial scale, is another part of the wastewater treatment design where modeling tools can help. Both detailed process simulators, such as GPS-X and SUMO,^52^ and simpler surrogate models can be used to accelerate scale-up by highlighting potential problems earlier in the design process and aiding in the design of experiments.

It is important to recognize the long lifespan of a WWTP, with core equipment typically lasting around 30 years.^53^ This means that decisions made today have long-term implications. At the same time, we need solutions that address current challenges while anticipating and adapting to future demands. Waiting until resource recovery becomes an urgent necessity is not an option, as reactive measures taken during a crisis are often too late. Given that new technologies usually take 20–30 years to mature and reach the market, it is imperative to invest now in future-proof innovations that will ensure long-term sustainability.

## Optimization of Wastewater Resource Recovery Using Machine Learning

To fully reveal the hotspots and trends of wastewater resource recovery from the perspective of research innovation, machine learning or, more specifically, data-driven methods can be utilized. These methods can be employed to screen all existing papers, reports, and patents,^54^ thereby speeding up the earliest stages of research and development.

In addition, machine learning can be used to facilitate WWTP design. While mechanistic models of wastewater systems are state-of-the-art, these models tend to be highly dimensional and complex, and so can be cumbersome to use in integrated process design.^55^ Machine learning surrogates, trained on data from first-principles process simulators,^56^ reduce the computational cost of process simulation by reducing model complexity, thereby accelerating computational process synthesis.^55^ While WWTP process data can be limited in quality,^57^ data reconciliation should be implemented to improve its usefulness.^58^ Moreover, standardization of data collection formats will be key to maximizing the value of these data to WWTP data scientists and process modelers using machine learning tools.

Machine learning is also starting to be applied to optimize WWTP operation, although it is usually based on physicochemical mechanisms rather than on microbiome features.^59,60^ For example, machine learning is generally advantageous for multiobjective optimization in wastewater treatment. Specifically, reinforcement learning (RL), a type of machine learning, is being used to simultaneously optimize energy consumption and effluent standards in an autopilot wastewater treatment plant (WWTP). A study with RL was conducted in a comparison within Benchmark Simulation Model No. 1 (BSM1). Results show that RL reduced energy use by 14.3% compared to BSM1 and outperformed advanced ammonia-based aeration control strategies.^61^ Integrating these machine learning techniques with digital twins is starting to be commercialized, such as in Veolia’s Hubgrade.^60^ When WWTP microbiome sequencing data are used, it is generally for detecting pathogens and antibiotic resistance genes,^62,63^ rather than for WWTP performance prediction or optimization. Proposals for better integration of WWTP microbiome sequencing data into WWTP operations do exist^64^ but are yet to be realized.

Increasing the use of computational aids in WWTP system design may accelerate the uptake of new resource recovery processes. Such tools can improve the ability of experts to consider a wide range of options, including less familiar ones, and model a larger number of trade-offs, which is invaluable when designs further from the status quo are being considered. New technological possibilities with or without microbial communities can be formulated as an optimization, with an objective and multiple constraints reflecting technical, operational, and legislative considerations.

## Implications

Rethinking WWTPs in light of new technological possibilities and novel insights into the potential and limitations of microbial communities should prioritize resource recovery and reuse.

This perspective highlights that the most impactful strategies lie in treating water to achieve potable reuse instead of producing a half-product to be discharged. In terms of energy recovery, routes to maximize the conversion of organics to methane and ammonia to hydrogen should be prioritized. Undoubtedly, artificial intelligence will be instrumental in accelerating progress along these lines.

Moreover, to achieve this, the following categorization has formulated:

- Bioprocesses with low prospects: nitrification, denitrification, biological phosphate removal, biological polymer production, N and P recovered from the water line to be used in agriculture.
- Bioprocesses to develop further: assimilation of C, N and P; anaerobic digestion to produce more methane; sorption of polar micropollutants on bioadsorbents; incorporation of recalcitrant organics in humus; bioleaching of sewage sludge ashes.
